# On The Randomized Schmitter Problem

**DOI:** 10.1007/s11009-021-09910-5

**Published:** 2021-11-08

**Authors:** Hansjörg Albrecher, José Carlos Araujo-Acuna

**Affiliations:** 1 Department of Actuarial Science, Faculty of Business and Economics, University of Lausanne, Switzerland and Swiss Finance Institute, Lausanne, Switzerland; 2 Department of Mathematics and Statistics, Institute of Mathematical Statistics and Actuarial Science, University of Bern, Bern, Switzerland

**Keywords:** Schmitter problem, Ruin theory, Ruin probability, Laplace transform, Stochastic ordering, Erlangization

## Abstract

We revisit the classical Schmitter problem in ruin theory and consider it for randomly chosen initial surplus level *U*. We show that the computational simplification that is obtained for exponentially distributed *U* allows to connect the problem to *m*-convex ordering, from which simple and sharp analytical bounds for the ruin probability are obtained, both for the original (but randomized) problem and for extensions involving higher moments. In addition, we show that the solution to the classical problem with deterministic initial surplus level can conveniently be approximated via Erlang(*k*)-distributed *U* for sufficiently large *k*, utilizing the computational advantages of the advocated randomization approach.

## Introduction

At the ASTIN Colloquium 1990 in Montreux, the Swiss actuary Hans Schmitter presented an algorithm for the exact evaluation of the ruin probability $\psi (u)$ of a Cramér-Lundberg surplus process for an insurance portfolio with initial surplus *u*, for the case when the claim amount distribution is discrete on a finite range (Schmitter (1990)). Also inspired by Bowers (1969), he then posed the following question: If the individual claims are known to have mean $\mu$ and variance $\sigma ^2,$ which claim size distributions minimize or maximize the ruin probability for a given *u*, respectively? I.e., the problems are

$$\begin{aligned} \begin{array}{cl} \text {min/max} &{} \psi (u) \\ \text {subject to} &{} X\text { is a non-negative random variable,} \\ &{} \text { with }\mathbb {E}(X)=\mu \text { and }\text {Var}(X)=\sigma ^2, \end{array} \end{aligned}$$

where *X* is the random variable describing the individual claim sizes. This problem was then further discussed by Brockett et al. (1991) and taken up in Kaas (1991), where it was also extended to the related problem of finding extremal values of stop-loss premiums for compound Poisson distributions with similar moment restrictions. Much later, De Vylder et al. (1997b); De Vylder and Marceau (1996) provided a numerical solution to the Schmitter problem based on a renewal equation that approximates the classical ruin model using a discrete time grid and partially solved the original problem in De Vylder et al. (1997a).

While on the basis of these contributions the problem can be considered as quite well understood, it was never solved in full generality. Correspondingly, despite the considerable time that has passed since then and the gradual shift of criteria for solvency considerations in insurance practice in the meantime, we would like to add an additional layer of complexity and understanding of the Schmitter problem in this paper by taking the perspective of a randomized initial surplus level.

Randomization as a principle has proven to be a very useful tool in risk theory leading to simpler expressions (see e.g. Albrecher et al. (2013), Ivanovs (2013)) or even unexpected identities (Albrecher and Ivanovs (2017)), but particularly also to considerable computational advantages (cf. Carr (1998), Asmussen et al. (2002), Albrecher and Goffard (2021)). The idea for the latter computational approach is to replace a deterministic quantity by a random variable with matching expected value, often with the advantage of smoothing the corresponding computational problem, leading to simpler and amenable expressions. In a final step, if possible the variance of that random variable is reduced considerably such that the resulting value can be an excellent approximation of the original computationally complex problem (“Erlangization”).

In our setting, we replace the deterministic initial surplus level *u* by an exponentially distributed random variable *U* with mean *u*. The expected value of the resulting ruin probability can then be expressed in terms of the (simpler) Laplace transform of the classical ruin probability under the Cramér-Lundberg model. At this level, analytical lower and upper bounds for the ruin probability in the Schmitter problem can then be established utilizing the strong results in the theory of *m*-convex orders obtained by Denuit et al. (1999, 1998) (see also Lefèvre et al. (2020) for a recent application of this ordering concept). In addition, the generality of the latter results in fact allows to give sharp upper and lower bounds for the ruin probability when more than two moments of the underlying claim size distribution are specified, which can be seen as an extension of the Schmitter problem that naturally narrows the gap between the upper and lower bound. For a comprehensive survey of stochastic orderings we refer to the monographs by Kaas et al. (1994), Shaked and Shanthikumar (1994, 2007) and Müller and Stoyan (2002). More recent treatments in a specifically actuarial context include Kaas et al. [Ch.7] (2008) and Asmussen and Steffensen [Ch.8] (2020).

Eventually, we are also interested in using these explicit expressions of the randomized model to approximate the classical situation of deterministic initial surplus level *u*. Developing the results further towards Erlang(*k*) distributed initial surplus, for increasing *k* (maintaining the expected value at *u*) this provides increasingly accurate approximations for the classical deterministic case, expressed through the explicit formulas of the randomized model.

The remaining paper is structured as follows. First, Section 2 recapitulates the model setting and summarizes relevant results from the existing literature. In Section 3 we then analyze the problem for an exponentially distributed initial surplus level *U*. We obtain an expression for the corresponding (expected) ruin probability in terms of the Laplace transform of the classical ruin probability in the Cramér-Lundberg model, and provide sharp lower and upper bounds for it when the claim size is bounded. We also provide corresponding bounds in the case of more than two pre-specified moments of the claim size distribution. Moreover, we illustrate the resulting interval for particular numerical parameters and place various concrete (truncated) claim size distributions within these bounds. In Section 4, we expand the randomization idea towards Erlang(*k*)-distributed initial surplus, and in the spirit of Asmussen et al. (2002) we approximate the ruin probability with deterministic surplus via Erlangization and Richardson extrapolation. We give numerical illustrations which show that the known and somewhat curious kinks in the graphs of the known optimal solutions of the classical Schmitter problem can be smoothly approximated with this randomization approach. In some cases, a small value of *k* is already sufficient for a good approximation, in others the value of *k* has to be quite considerable. Section 5 concludes.

## Preliminaries and Previous Results

Consider the classical Cramér-Lundberg model with surplus process

$$C(t)=u+ct -S(t),$$

at time $t\ge 0$, where *u* is the initial surplus level. Here, $S(t) = X_1 + \cdots + X_{N(t)}$ denotes the aggregate claims up to time *t*, where the number of claims $\{N(t);\, t\ge 0\}$ up to time *t* refers to a homogeneous Poisson process with rate $\lambda >0$ and the claim sizes $X_i, i=1,2,\ldots ,$ are independent and identically distributed random variables with distribution function $F_{X}$ and expected value $\mathbb {E}(X_1)=\mu$, independent of $\{N(t);\, t\ge 0\}$. We assume that all moments of $X_1$ exist. The premium income per unit of time is $c = (1+\theta )\lambda \mu$, where $\theta >0$ is the safety loading. Define the associated aggregate loss process as $R(t) = S(t) - ct,$ for $t\ge 0.$ The probability $\psi (u)$ of ultimate ruin is the probability that the surplus process *C*(*t*) ever drops below zero,

$$\psi (u) = \mathbb {P}\left( \inf _{t\ge 0} C(t)<0 \right) = \mathbb {P}\left( \sup _{t\ge 0} R(t) > u \right) .$$

The maximal aggregate loss $L = \sup _{t\ge 0} R(t)$ can be decomposed as the sum of ladder heights, i.e. as the sum of the amounts by which record lows (here denoted by $L_1, L_2, \ldots$) in the insurer’s surplus *C*(*t*) appear. Furthermore, the distribution of the $L_i\,(i=1, 2, \ldots )$ is given by the integrated tail distribution $F_{L_i}(x) = \mu ^{-1}\int _0^x (1-F_X(z))dz$, $x>0.$ It is well known that $\psi (u)$ is given explicitly by the Pollaczeck-Khinchine formula

1 $$\begin{aligned} \psi (u) = \frac{\theta }{1+\theta }\sum _{k=0}^{\infty } \left( \frac{1}{1+\theta }\right) ^k (1-{F}_{L_i}^{*k}(u)), \end{aligned}$$

where ${F}_{L_i}^{*k}$ denotes the *k*-fold convolution of the ladder height distribution (see e.g. Asmussen and Albrecher [Th.IV.2.1] (2009)). The latter expression shows that *L* is a compound geometric random variable and may be written as $L = \sum _{k=1}^M L_i,$ with *M* being the number of ladder heights. It is easy to see that *M* has a geometric distribution with parameter $\psi (0) = 1/(1+\theta )$ (see, for example Asmussen and Albrecher [Cor.IV.3.1] (2009)). The Laplace transform of (1) is well-known to be

2 $$\begin{aligned} \widehat{\psi }(s) = \int _0^{\infty } e^{-su}\psi (u)du = \frac{1}{s} - \frac{c-\lambda \mu }{cs-\lambda (1-M_X(-s)),} \end{aligned}$$

where $M_X(-s)=\int _0^\infty e^{-sx} dF_X(x)$ is the Laplace-Stieltjes transform of *X* (cf. Rolski et al. [Th.5.3.3] (2009) or Asmussen and Albrecher [Cor.IV.3.4] (2009)).

For the case when the claim amount distribution has discrete support $\{x_1, x_2, \ldots , x_m\}$ (with probabilities $p_1, p_2, \ldots , p_m$), Schmitter (1990) gave an explicit expression to compute $\psi (u)$ in the form

$$\begin{aligned} \psi (u) =1-\frac{\theta }{1+\theta } \sum _{l_1,\cdots ,l_m}(-z_m)^{l_1+\cdots l_m}\text {e}^{z_m}\prod _{j=1}^{m}\frac{p_j^{l_j}}{l_j!}, \end{aligned}$$

where $$z_m=(u-l_1\cdot x_1 - \cdots - l_m\cdot x_m)_{+}/\mu \cdot (1+\theta )$$ and $$z_{+} = \max (z, 0).$$

In the context of the Schmitter problem, 2-point distributions for the claim size play a special role. If *X* assumes the values $x_1$ with probability *p* and $x_2>x_1$ with probability $1-p$, then for fixed mean $\mu >0$ and variance $\sigma ^2>0$ we simply have

$$\begin{aligned} \mu = x_1\cdot p + x_2\cdot (1-p) ~ \text { and }~ \sigma ^2 + \mu ^2 = x_1^2\cdot p + x_2^2\cdot (1-p), \end{aligned}$$

or correspondingly

3 $$\begin{aligned} x_1=\mu -\frac{\sigma ^2}{x_2-\mu },\, x_2=\mu +\frac{\sigma ^2}{\mu -x_1} \text {and}\, p = \frac{\sigma ^2}{\sigma ^2 + (\mu -x_1)^2}, \end{aligned}$$

Notice that $x_2$ is increasing in $x_1$. Moreover, one has the relationships

$$\begin{aligned} \frac{\sigma ^2}{\mu ^2+\sigma ^2}\le p< 1, 0\le x_1< \mu , \text { and } \frac{\mu ^2+\sigma ^2}{\mu }\le x_2 \end{aligned}$$

(see e.g. Kaas et al. [Ch 10.2] (1994)). If we additionally assume that $X\in [0, b]$, naturally $x_2 \le b,$ and we have $0 \le \mu \le b$ and $0\le \sigma ^2 \le \mu (b-\mu )$. The following two extremal cases will be particularly relevant later. Namely, $X=\{0,\,0^* := (\mu ^2+\sigma ^2)/\mu \}$ and so $p=\sigma ^2/(\sigma ^2+\mu ^2)$ and $X =\{b^* :=\mu +\sigma ^2/(\mu -b),\,b\}.$ In here, $x^*$ denotes the function that assigns to *x* the unique real number such that the random variable $X = \{x, x^*\}$ has mean $\mu$ and variance $\sigma ^2.$ Note that if *b* is not bounded, then as $x_1\uparrow \mu$, $p\uparrow 1$ and $x_2\rightarrow \infty$; while the probability mass at $x_2$ becomes arbitrarily small, it significantly contributes to the variance.

For any non-negative loss variable *X*, the stop-loss premium $\pi _X$ is defined by

$$\pi _X(d) = \mathbb {E}((X-d)_{+}) = \int _d^{\infty } (1-F_X(z))dz, ~ \text { for } d\ge 0.$$

Note that there is a one-to-one relation between the integrated tail distribution of *X* and its stop-loss premium, namely $F_{L_i}(z) = 1-\pi _X(z)/\mu .$ One important concept in the theory of risk ordering is the stop-loss order. Concretely, a random variable *X* is said to be less risky than another random variable *Y* in stop-loss order ($X\le _{\text {sl}} Y$) if $\pi _X(d) \le \pi _Y(d)$ for all retentions $d\ge 0$ (it is equivalent to increasing convex ordering, cf. Shaked and Shanthikumar (2007)). The problem of finding bounds for stop-loss premiums is a classical topic in actuarial science, see for example Bühlmann et al. (1977), Kaas and Goovaerts (1986) and Steenackers and Goovaerts (1991). For a study of the relation between stop-loss premiums and their associated ruin probabilities as well as general upper bounds for both stop-loss premiums and ruin probabilities see Cai and Garrido (1998) and the references therein.

A consequence of the above concept is that if for two Cramér-Lundberg risk processes with equal premium per unit of time and claim intensity parameter, but different claim sizes, say *X* and *Y*, with $X\le _{\text {sl}} Y$ we have $\psi _X(u) \le \psi _Y(u)$ for all $u\ge 0$ (see Kaas et al. [Ch.8.2,Th.2.1] (1994)). Correspondingly, the Schmitter problem may be seen as being reduced to finding extremal distributions in the stop-loss order in the class of random variables in [0, *b*] with mean $\mu$ and variance $\sigma ^2$. However, as pointed out in Brockett et al. (1991) there are no extremal distributions in terms of stop-loss order in such a class.

Nevertheless one can construct stop-loss transforms in the corresponding range (bounded or not) with the given mean, but with minimal variance, larger than the given one. For two given moments, the latter is achieved by constructing a polynomial of degree 2 above the function $(X-d)_{+}$ which is tangent to this function in 2 points. The abscissas of these points will be the mass points. For a comprehensive description of this construction see Kaas et al. [Ch.10] (1994). In the following we briefly state its main consequences.

For unbounded *X* with mean $\mu$ and variance $\sigma ^2$, the maximal stop-loss premium at fixed retention *d* is attained by a random variable *Z* with support $\{r,r^*\}$, where $r,r^*=d\mp \sqrt{(\mu -d)^2+\sigma ^2}$, and from (3) then $\mathbb {P}(X=r) = \sigma ^2/(\sigma ^2 + (\mu -r)^2)$. Note that $\{r,r^*\}$ is the 2-point support that has *d* in the middle. If $X\in [0,b]$, this 2-point distribution still gives an upper bound, but it is no longer always sharp. Theorem 2.3 in Kaas et al. [Ch.10] (1994) provides a sharp upper bound for stop-loss premiums for $X\in [0,b]$. For given retention *d*, the maximal stop-loss premium is attained by the distribution with the mass points

$$\begin{aligned} \{0,\,0^*\} \quad&\text {if}\; 0\le d \le \frac{1}{2} 0^*,\\ \{r,\,r^*\} \quad&\text {if}\;\frac{1}{2} 0^*\le d \le \frac{1}{2} (b+b^*),\\ \{b^*,\, b\} \quad&\text {if}\;\frac{1}{2} (b+b^*)\le d \le b \end{aligned}$$

with the notation introduced before. However, these results do not provide an upper bound for the ruin probability in the Schmitter problem, because it is not the same extremal distribution across all values of *d*, but the latter would be needed to bequeath the dominance in terms of the stop loss premium from the integrated tail to all its convolutions in (1). However, Kaas (1991) showed that if *X* has lower stop-loss premiums than *Y* on the interval [0, *u*], then the same property holds for compound sums with *N* terms of these random variables respectively, and ruin probabilities with an initial surplus *u* are lower for *X* than for *Y*. That is, for values of *u* smaller than $\frac{1}{2}0^*$, the ruin probability is maximized by the 2-point claim random variable $X = \{0, 0^*\}.$ Consequently, in terms of the upper bound the Schmitter problem is solved for small values of the initial surplus *u*.

De Vylder and Marceau (1996) and De Vylder et al. (1997b) provided numerical solutions to the problem based on a renewal equation in a discretized risk model. By restricting to lattice distributions, they used the method of linear combinations (see also Kaas et al. [Sec.3] (1992)) to obtain optimal solutions to the problem. They noted that for $u\gg b$ the maximal ruin probability was given by the 2-point claim random variable $X = \{b^*, b\}.$ In fact, De Vylder et al. (1997a) then proved that there exists a constant $c>0$ such that for all $u\ge c$ the maximal ruin probability is given by that 2-point claim random variable. However, the concrete value of *c* as well as the optimal result for intermediate values of *u* seem to still not be settled up to this day.

The minimal stop-loss premium for risks *X* with mean $\mu$ is given by $(\mu -d)_+$ for all retentions $d\ge 0$, i.e. it is attained by the defective random variable *Z* concentrated at $\mu ,$ implying $Z \le _{\text {sl}} X$ and therefore $\psi _Z(u) \le \psi _X(u)$ for all *u*. However, *Z* does not fulfill the variance constraint, so that this is not a valid solution to the Schmitter problem. It does provide a general lower bound for its solution though, and for unbounded *X* the variance constraint can then be satisfied by adding an $\varepsilon \,(\downarrow 0)$ mass at infinity, see also Asmussen and Albrecher [Cor. IV.8.4] (2009).

## Exponentially Distributed Initial Surplus

Let us now replace the deterministic initial surplus *u* by a random variable *U* that has an exponential distribution with parameter $s>0$. The redefined surplus process then is

$$C^R(t) = U + ct - S(t),\text { }t\ge 0,$$

where *c* and *S*(*t*) are defined as in the classical ruin model, and *U* is independent of *S*(*t*). Using the convenient fact that this choice of *U* simply puts us in the framework of Laplace transforms, due to (2) the ruin probability $\psi _U(s):=\mathbb {P}(C^R(t)<0$ for some $t>0$) is then given by

4 $$\begin{aligned} \psi _U(s) := \mathbb {E}(\psi (U)) =\int _{0}^{\infty }\psi (u)s e^{-su}du = s\cdot \widehat{\psi }(s) = 1-s\cdot \frac{c-\lambda \mu }{cs-\lambda (1-M_X(-s))}. \end{aligned}$$

**Fig. 1:**
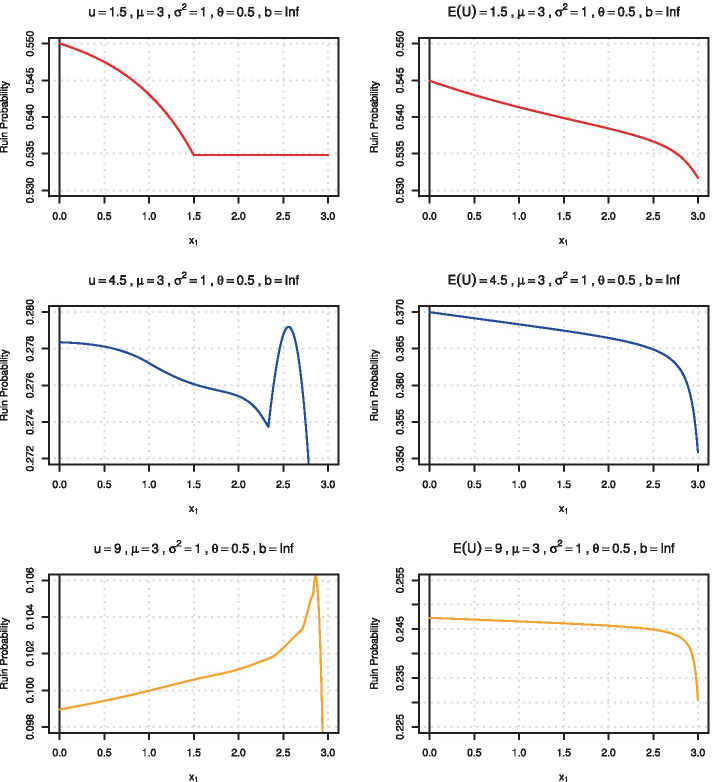
Ruin probabilities as a function of $x_1$ for $\mu =3$, $\sigma ^2=1$, $\theta =0.5$ for the three deterministic surplus levels $u=1.5,4.5,9$ (*left column*) and the randomized counterpart with the same expected initial surplus level (*right column*)

Since the randomization of the initial surplus corresponds to a probability-weighted averaging over situations with deterministic surplus, it is clear that this step leads to a smoothing of the ruin probability shape. Figure 1 compares the ruin probabilities $\psi (u)$ for deterministic surplus $u = \{1.5, 4.5, 9.0\}$ and $\theta = 0.5$ (the parameters from Kaas [Fig. 1] (1991)) with the corresponding randomized quantities of the same expected initial surplus $\mathbb {E}(U)=1/s=u$ for 2-point distributions with given mean $\mu = 3$ and variance $\sigma ^2 = 1$. One observes that the sensitivity w.r.t. the choice of the only free parameter $x_1$ is substantially different, and the somewhat curious shape change for increasing *u* from the classical deterministic case is indeed evened out.

Let us now look at the randomized and extended Schmitter problem

$$\begin{aligned}&\text {min/max}&\psi _U(s) \\&\text {subject to}&\mathbb{E}(X^k)&= \mu _k, \text { for } k = 1, 2, \ldots , m\end{aligned}$$

with possibly more than two fixed moments of the claims size distribution. Inspired by Kaas (1991), using the maximal aggregate loss *L* and assuming that the moments of the claim size are finite, one can express the ruin probability in terms of the claim size moments, namely

$$\begin{aligned} \psi _U(s)&=\int _{0}^{\infty }\psi (u)s e^{-su}du = \sum _{k=0}^{\infty } (-1)^k\frac{s^{k+1}}{k!} \int _{0}^{\infty }u^k \psi (u) du\\&= \sum _{k=0}^{\infty }(-1)^k\frac{s^{k+1}}{k!} \int _{0}^{\infty }u^k \mathbb {P}(L > u) du = \sum _{k=1}^{\infty } (-1)^{k-1}\frac{s^k}{k!} \,\mathbb {E}(L^k)\\&= \sum _{k=1}^{\infty } (-1)^{k-1}\frac{s^k}{k!} \mathbb {E}\left( \mathbb {E}\left( \sum _{l_1 + l_2 + \cdots + l_M = k} {k \atopwithdelims ()l_1, l_2, \ldots , l_M} \prod _{j=1}^M L_j^{l_j} \Big \vert M \right) \right) . \end{aligned}$$

The first four terms of this series are given by

$$\begin{aligned} E(L) &{}= \mathbb {E}(M)\mathbb {E}(L_1) = \frac{1}{2\theta \mu }\mathbb {E}(X^2) \\ E(L^2) &{}= \mathbb {E}(M)\mathbb {E}(L^2_1) + \mathbb {E}(M(M-1))\mathbb {E}^2(L_1) = \frac{1}{3\theta \mu }\mathbb {E}(X^3) + \frac{1}{2\theta ^2\mu ^2}\mathbb {E}(X^2)\\ E(L^3) &{}= \frac{1}{4\theta \mu }\mathbb {E}(X^4) + \frac{1}{\theta ^2\mu ^2}\mathbb {E}(X^3)\mathbb {E}(X^2) + \frac{3}{4\theta ^3\mu ^3}\mathbb {E}^3(X^2) \\ E(L^4) &{}= \frac{1}{5\theta \mu }\mathbb {E}(X^5) + \frac{1}{\theta ^2 \mu ^2}\mathbb {E}(X^4)\mathbb {E}(X^2) + \frac{2}{3\theta ^2 \mu ^2}\mathbb {E}^2(X^3) \\ {} &{}\qquad \qquad + \frac{1}{6\theta ^3\mu ^3}\mathbb {E}(X^3)\mathbb {E}^2(X^2) + \frac{3}{2\theta ^4 \mu ^4}\mathbb {E}^4(X^2) \end{aligned}$$

Hence, if the first *m* moments of *X* are given, then one can approximate

5 $$\begin{aligned} \psi _U(s) \approx s\,\mathbb {E}(L) - \frac{s^2}{2}\mathbb {E}(L^2) + \cdots + (-1)^{m-1}\frac{s^{m}}{m!} \mathbb {E}(L^m) \end{aligned}$$

and investigate the behavior with respect to the highest moment. For example, for $m=2$

$$\begin{aligned} \psi _U(s) \approx s\,\frac{\sigma ^2 + \mu ^2}{2\theta \mu } - \frac{s^2}{6\theta \mu } \mathbb {E}(X^3) - s^2\frac{(\sigma ^2 + \mu ^2)^2}{4\theta ^2\mu ^2}. \end{aligned}$$

Therefore, distributions with large third moment will make $\psi _U(s)$ small and vice versa. For 2-point distributions, a simple calculation shows that, $\frac{\partial }{\partial x_1} \mathbb {E}(X^3) = \sigma ^2 + \left( \frac{\sigma ^2}{\mu - x_1}\right) ^2 > 0$ and $\frac{\partial ^2}{\partial x_1^2} \mathbb {E}(X^3) = 2\frac{\sigma ^2}{(\mu - x_1)^3} > 0.$ Thus, for $x_1\in [0, \mu )$, its third moment is increasing and convex, so the maximum will be at $x_1 = 0$ and the minimum at $x_1 \rightarrow \mu .$ In fact, for deterministic surplus and 2-point distributions, Kaas (1991) argued that as $\int _0^{\infty } \psi (u) du = \mathbb {E}(L)$ does not depend on $x_1$ and $\int _0^{\infty } u\psi (u) du = \mathbb {E}(L^2)$ increases linearly with the third moment of the claim distribution, so that for small *u*, the ruin probability will be large for $x_1=0.$

While these considerations are intuitive, from (4) it becomes clear that for the extremal values of the randomized ruin probability it suffices to minimize (maximize) the Laplace transform of the individual claim sizes, i.e. to find extremal random variables in the Laplace transform order. The Laplace transform order has been introduced by Rolski and Stoyan (1976) to compare waiting times in queuing theory. In actuarial science, Denuit (2001) studied both univariate and multivariate versions of the Laplace transform order and gave several actuarial applications. We can now give sharp bounds for the randomized Schmitter problem for two given moments.

### Proposition 3.1

Let *X* be a non-negative random variable with mean $\mu$ and variance $\sigma ^2$. Then

$$\begin{aligned} 1-s\cdot \frac{c-\lambda \mu }{cs-\lambda (1-e^{-s\mu })} \le \psi _U(s) \le 1-s\cdot \frac{c-\lambda \mu }{cs-\lambda (1-\frac{\sigma ^2}{\sigma ^2+\mu ^2}- \frac{\mu ^2}{\sigma ^2+\mu ^2}e^{-s(\mu +\sigma ^2/\mu )})} . \end{aligned}$$

### Proof

Note that $e^{-s\mu }$ is the Laplace transform of a random variable *Y* degenerate at $\mu .$ Moreover, $\frac{\sigma ^2}{\sigma ^2+\mu ^2}+ \frac{\mu ^2}{\sigma ^2+\mu ^2}e^{-s(\mu +\sigma ^2/\mu )}$ is the Laplace transform of a random variable *Z* with mean $\mu$, variance $\sigma ^2$ and such that $\mathbb {P}(Z = 0) = 1-\mathbb {P}(Z = (\mu ^2+\sigma ^2)/\mu ) = \frac{\sigma ^2}{\sigma ^2 + \mu ^2}$. Therefore, as maximizing the Laplace transform of the individual claim sizes minimizes $\psi _U(s)$ and vice versa, it suffices to show that $Y \le _{\text {Lt}} X \le _{\text {Lt}} Z.$ The proof of the latter can be found in Shaked and Shanthikumar [Ch. 5, Theorem 5.A.21] (2007). $\square$

It is worth noticing that the distribution maximizing the randomized ruin probability coincides with the 2-point distribution that maximizes the ruin probability under deterministic surplus for small values of *u*. This is rather intuitive, since $\psi _U(s)$ is a weighted average of $\psi (u)$ with a lot of weight for small values of *u*.

If more moments of the claim size *X* in [0, *b*] are specified, then one can obtain tighter upper and lower bounds for the randomized ruin probability. In view of (4), this reduces to the derivation of bounds for the Laplace transform of *X* in the moment space $\mathcal {B}_S([0,b]; \mu _1, \mu _2, \ldots , \mu _m)$ of all risks *X* with range [0, *b*] such that $\mathbb {E}(X^k) = \mu _k$ for $k = 1, 2, \ldots , m.$ Fortunately, our context fits exactly into the framework of Denuit et al. (1999, 1998) who constructed lower and upper stochastic bounds for a given set of risks using *m*-convex stochastic orders. More precisely, consider the class $\mathcal {M}_{m-cx}$ of all functions $\phi : [0, b] \rightarrow \mathbb {R}$ whose $(m+1)$-th derivative $\phi ^{(m+1)}(x)$ exists and is non-negative, for all $x\in [0, b]$, or which are limits of sequences of functions whose $(m+1)$-th derivative is continuous and non-negative on [0, *b*]. Define the partial order relation $\le _{m-cx}$ among elements in $\mathcal {B}_S$ as

6 $$\begin{aligned} X\le _{m-cx}Y \text { if and only if } \mathbb {E}(\phi (X)) \le \mathbb {E}(\phi (Y)) \text { for all functions } \phi \in \mathcal {M}_{m-cx}, \end{aligned}$$

provided the expectations exists. It is then possible to determine two discrete risks $X^{(m)}_{\min }$ and $X^{(m)}_{\max },$ in $\mathcal {B}_S([0,b]; \mu _1, \mu _2, \ldots , \mu _m)$ with probability masses depending on the moment set $(\mu _1, \mu _2, \ldots , \mu _m)$ such that

7 $$\begin{aligned} X^{(m)}_{\min } \le _{m-cx} X \le _{m-cx} X^{(m)}_{\max } \text { for all } X\in \mathcal {B}_S. \end{aligned}$$

Explicit descriptions for the distribution functions of the extrema up to $m = 4$ are obtained in Denuit et al. [Table 1, Table 2] (1999). Moreover, the latter reference also provided the extrema with respect to the order $\le _{m-cx}$ when not only the first $m-1$ moments and the support are given, but also when the density function of *X* is known to be unimodal with a known mode.^1^

### Proposition 3.2

Let $X\in [0, b], ~b>0,$ with moments $(\mu _1, \mu _2, \ldots , \mu _m).$ Then,

8 $$\begin{aligned} &M_{X^{(m)}_{\max }}(-s) &\le M_{X}(-s)& \le M_{X^{(m)}_{\min }}(-s), \text { for m+1 odd} \\& M_{X^{(m)}_{\min }}(-s) &\le M_{X}(-s)& \le M_{X^{(m)}_{\max }}(-s), \text { for m+1 even,} \end{aligned}$$

which can then be translated to bounds for $\psi _U(s).$

### Proof

Since $\phi (x) = (-1)^{m+1} e^{-sx}$ belongs to $\mathcal {M}_{m-cx}$ for $s > 0,$ the claim follows from (6) and (7).

The bounds for the Laplace transform using $m-$convex risks were already described in Denuit et al. (2000), extending earlier works of Eckberg (1977), Whitt (1983) and Lefèvre et al. (1986). In particular, Eckberg (1977) derived bounds for the Laplace transform up to the third moment using the theory of Chebychev systems and applied the bounds to problems in queuing and traffic theory. Moreover, the latter reference provides bounds for the case where no upper bound is known. We would also like to mention that, closely related to the theory of *m*-convex stochastic orders, using Markov-Krein theory and the theory of Chebychev systems, Brockett and Cox (1984, 1985) obtained similar upper and lower bounds for the expected value of a function of some random variable with given moments. Also, De Vylder (1982, 1983), De Vylder and Goovaerts (1982), Kaas and Goovaerts (1986) and Hürlimann (1998) examined related bounding problems.

Using (8) we can give explicit bounds for the ruin probability with exponentially distributed initial surplus in terms of the given parameters. For reference, we restate here the respective bounds given in Denuit et al. [Table 1, Table 2] (1999)) in terms of ruin probabilities when up to 4 moments of *X* are given:

Case $m=1$. If $\mu _1$ is given, then $X^{(1)}_{\min }$ is a random variable degenerate at $\mu _1$, and

$$\begin{aligned} X^{(1)}_{\max }&= {\left\{ \begin{array}{ll} 0 &{} \text { with } p = 1 - \frac{\mu _1}{b}, \\ b &{} \text { with } 1-p = \frac{\mu _1}{b}. \end{array}\right. } \end{aligned}$$

Therefore,

$$\begin{aligned} \psi ^{\min }_U(s)&= 1-s\cdot \frac{c-\lambda \mu _1}{cs-\lambda (1-e^{-s\mu _1})}, \\ \psi ^{\max }_U(s)&= 1-s\cdot \frac{c-\lambda \mu _1}{cs-\frac{\lambda \mu _1}{b}(1-e^{-sb})} . \end{aligned}$$

Case $m=2$. If $\mu _1$ and $\mu _2$ are given, then

$$\begin{aligned} X^{(2)}_{\min } = {\left\{ \begin{array}{ll} 0 &{} \text { with } p = 1-\frac{\mu _1^2}{\mu _2},\\ \frac{\mu _2}{\mu _1} &{} \text { with } 1-p = \frac{\mu _1^2}{\mu _2}, \end{array}\right. } \qquad X^{(2)}_{\max } = {\left\{ \begin{array}{ll} \frac{b\mu _1 - \mu _2}{b-\mu _1} &{} \text { with } p =\frac{(b-\mu _1)^2}{(b-\mu _1)^2 + \mu _2 - \mu _1^2},\\ b &{} \text { with } 1-p = \frac{\mu _2 - \mu _1^2}{(b-\mu _1)^2 + \mu _2 - \mu _1^2}. \end{array}\right. } \end{aligned}$$

In this case, it can be seen that

$$\begin{aligned} \psi ^{\min }_U(s)&= 1-s\cdot \frac{c-\lambda \mu _1}{cs-\lambda (1-\frac{(b-\mu _1)^2}{\sigma ^2+(b-\mu _1)^2} e^{-s(\mu _1-\sigma ^2/(b-\mu _1))}-\frac{\sigma ^2}{\sigma ^2+(b-\mu _1)^2} e^{-sb})}, \\ \psi ^{\max }_U(s)&= 1-s\cdot \frac{c-\lambda \mu _1}{cs-\frac{\lambda \mu _1^2}{\sigma ^2+\mu _1^2}(1-e^{-s(\mu _1+\sigma ^2/\mu _1)})} . \end{aligned}$$

Note that for $b\rightarrow \infty$ the above expressions indeed converge to the bounds given in Proposition 3.1.

Case $m=3$. If $\mu _1,$ $\mu _2$ and $\mu _3$ are given, then

$$\begin{aligned} X^{(3)}_{\min }&= {\left\{ \begin{array}{ll} r_+ = \frac{\mu _3 - \mu _1\mu _2 + \sqrt{(\mu _3 - \mu _1\mu _2)^2 - 4\sigma ^2(\mu _1\mu _3 - \mu _2^2)}}{2\sigma ^2} &{} \text { with } p = \frac{\mu _1 - r_{-}}{r_{+}- r_{-}},\\ r_- = \frac{\mu _3 - \mu _1\mu _2 - \sqrt{(\mu _3 - \mu _1\mu _2)^2 - 4\sigma ^2(\mu _1\mu _3 - \mu _2^2)}}{2\sigma ^2} &{} \text { with } 1-p = 1-\frac{\mu _1 - r_{-}}{r_{+}- r_{-}}, \end{array}\right. } \\ X^{(3)}_{\max }&= {\left\{ \begin{array}{ll} 0 &{} \text { with } p_3 =1-p_1-p_2,\\ \frac{\mu _3 - b\mu _2}{\mu _2 - b\mu _1} &{} \text { with } p_1 = \frac{(\mu _2 - b\mu _1)^3}{(\mu _3 - b\mu _2)(\mu _3 - 2b\mu _2 + b^2\mu _1)},\\ b &{} \text { with } p_2 = \frac{\mu _1\mu _3 - \mu _2^2}{b(\mu _3 - 2b\mu _2 + b^2\mu _1)}. \end{array}\right. } \end{aligned}$$

Then, the bounds for the ruin probability are given by

$$\begin{aligned} \psi ^{\min }_U(s)&= 1-s\cdot \frac{c-\lambda \mu _1}{cs-\lambda \left( 1-\left( 1-\frac{\mu _1 - r_{-}}{r_{+}- r_{-}}\right) e^{-sr_{-}}-\left( \frac{\mu _1 - r_{-}}{r_{+}- r_{-}} \right) e^{-sr_{+}}\right) },\\ \psi ^{\max }_U(s)&= 1-s\cdot \frac{c-\lambda \mu _1}{cs-\lambda \left( p_1\left( 1- e^{-s\frac{\mu _3 - b\mu _2}{\mu _2-b\mu _1}}\right) + p_2\left( 1-e^{-sb}\right) \right) }. \end{aligned}$$

Case $m=4$. If $\mu _1$ and up to $\mu _4$ are given, then

$$\begin{aligned} X^{(4)}_{\min }&= {\left\{ \begin{array}{ll} 0 &{} \text { with } 1 -p_{+} - p_{-},\\ t_{+} = \frac{\mu _1\mu _4 - \mu _2\mu _3 + \sqrt{(\mu _1\mu _4 - \mu _2\mu _3)^2 - 4(\mu _1\mu _3 - \mu _2^2)(\mu _2\mu _4 - \mu _3^2)}}{2(\mu _1\mu _3 - \mu ^2_2)} &{} \text { with } p_{+} = \frac{\mu _2 - t_{-}\mu _1}{t_{+}(t_{+} - t_{-})},\\ t_{-} = \frac{\mu _1\mu _4 - \mu _2\mu _3 - \sqrt{(\mu _1\mu _4 - \mu _2\mu _3)^2 - 4(\mu _1\mu _3 - \mu _2^2)(\mu _2\mu _4 - \mu _3^2)}}{2(\mu _1\mu _3 - \mu ^2_2)} &{} \text { with } p_{-} = \frac{\mu _2 - t_{+}\mu _1}{t_{-}(t_{-} - t_{+})}. \end{array}\right. } \\ X^{(4)}_{\max }&= {\left\{ \begin{array}{ll} z_{+}= \frac{(\mu _1 - b)(\mu _4 - b\mu _3)-(\mu _2 - b\mu _1)(\mu _3 - b\mu _2)+\sqrt{\rho }}{2\left( (\mu _1 - b)(\mu _3 - b\mu _2)-(\mu _2 - b\mu _1)^2\right) } &{} \text { with } q_{+} = \frac{\mu _2 - (b+z_{-})\mu _1 + bz_{-}}{(z_{+} - z_{-})(z_{+}-b)},\\ z_{-}= \frac{(\mu _1 - b)(\mu _4 - b\mu _3)-(\mu _2 - b\mu _1)(\mu _3 - b\mu _2)-\sqrt{\rho }}{2\left( (\mu _1 - b)(\mu _3 - b\mu _2)-(\mu _2 - b\mu _1)^2\right) } &{} \text { with } q_{-} = \frac{\mu _2 - (b+z_{+})\mu _1 + bz_{+}}{(z_{-} - z_{+})(z_{-}-b)},\\ b &{} \text { with } 1-q_{+} - q_{-}. \end{array}\right. } \end{aligned}$$

Here,

$$\begin{aligned} \rho :=&\left( (\mu _1 - b)(\mu _4 - b\mu _3) - (\mu _2-b\mu )(\mu _3 - b\mu _2)\right) ^2 \\&\quad -4\left( (\mu _1 - b)(\mu _3 - b\mu _2)-(\mu _2 - b\mu _1)^2 \right) \left( (\mu _2 - b\mu _1)(\mu _4 - b\mu _3)-(\mu _3 - b\mu _2)^2\right) \end{aligned}$$

As can easily be verified,

$$\begin{aligned} \psi ^{\min }_U(s)&= 1-s\cdot \frac{c-\lambda \mu _1}{cs-\lambda \left( 1-q_{+} e^{-sz_{+}}- q_{-}e^{-sz_{-}}-(1-q_{+}-q_{-})e^{-sb}\right) },\\ \psi ^{\max }_U(s)&= 1-s\cdot \frac{c-\lambda \mu _1}{cs-\lambda \left( p_{+}(1- e^{-st_{+}})+ p_{-}(1-e^{-st_{-}})\right) }. \end{aligned}$$

### Numerical Illustrations

De Vylder [Sec.II, Ch.3] (1996) gives conditions for the class of all vectors $(\mu _1, \mu _2, \ldots , \mu _m)\in \mathbb {R}^m$ such that $\mathcal {B}_S([0,b]; \mu _1, \mu _2, \ldots , \mu _m)$ is not empty. In Denuit et al. [Sec.4.1] (1999), this class of all possible moment sequences is denoted by $\mathcal {D}_m([0,b]).$ Moreover, they provided expressions for the topological interior, $\mathcal {D}_m\circ ([0,b])$, of $\mathcal {D}_m([0,b])$ up to $m = 4.$ For completeness we cite the three cases relevant for our applications here, namely:

$$\begin{aligned} \mathcal {D}_1\circ ([0,b])&= \{\mu _1\in \mathbb {R}\vert 0< \mu _1< b\}, \\ \mathcal {D}_2\circ ([0,b])&= \{(\mu _1, \mu _2) \in \mathbb {R}^2\vert \mu _1 \in \mathcal {D}_1\circ ([0,b]) \text { and } \mu _1^2< \mu _2< \mu _1 b\},\\ \mathcal {D}_3\circ ([0,b])&= \{(\mu _1, \mu _2, \mu _3) \in \mathbb {R}^3\vert (\mu _1, \mu _2) \in \mathcal {D}_2\circ ([0,b]) \text { and } \\&\frac{\sigma ^2}{\mu _1}(\sigma ^2 - \mu _1^2) -2\mu _1^3 + 3\mu _1\mu _2< \mu _3 < (b-\mu _1)\sigma ^2 -\frac{\sigma ^4}{b-\mu _1}-2\mu _1^3 + 3\mu _1\mu _2\}. \end{aligned}$$

**Fig. 2:**
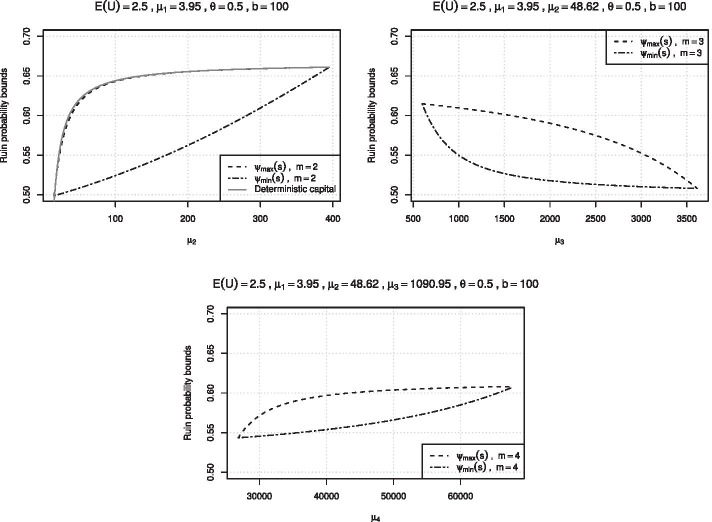
Sharp bounds for the randomized ruin probability $\psi _U(2/5)$, considered as a function of $\mu _2,\,\mu _3$ and $\mu _4$ respectively

Figure 2 depicts the sharp bounds for the ruin probability with $b=100,$ $\theta = 0.5$ and $s = 2/5,$ i.e. $\mathbb {E}(U) = 2.5.$ The upper left figure shows the bounds for $\mu _1 = 3.95$ as a function of $\mu _2$ satisfying $(\mu _1, \mu _2)\in \mathcal {D}_2\circ ([0,b]).$ For this case, we also know the upper bound solution of the Schmitter problem with deterministic surplus and we can compare the two. It turns out that the upper bounds of the randomized and the deterministic case are remarkably close. The upper right figure shows the sharp ruin probability bounds for three given moments as a function of $\mu _3$ satisfying $(3.95, 48.62, \mu _3)\in \mathcal {D}_3\circ ([0,b]).$ As remarked in the previous section, once sees that the ruin probability decreases with increasing third moment. As expected, the bounds are tighter as the knowledge of the second moment is incorporated. Finally, the graph at the bottom depicts the bounds of the generalized randomized Schmitter problem for given four moments of *X* as a function of $\mu _4$, leading to yet tighter bounds. Note that in this numerical illustration the values of the first three moments were selected in such a way that one finds a feasible set of distribution parameters for all of the distributions in the following numerical illustration.

In order to illustrate the performance of the bounds and how they improve with the addition of moment information, we explicitly calculate $\psi _U(s)$ for some chosen claim size distribution in each case, suitably truncated so that it fits into the model setup:

- Case $m=1.$ Truncated Exponential ($\lambda$) model with distribution function given by
  $$\begin{aligned} F_X(x) = \frac{1-e^{-\lambda x}}{1-e^{-\lambda b}}, ~ 0<x\le b, ~ \lambda >0, \end{aligned}$$
  and Laplace transform
  $$\begin{aligned} M_X(-s) = \frac{\lambda }{\lambda + s} \frac{1-e^{-(\lambda +s)b}}{1-e^{-\lambda b}}. \end{aligned}$$
- Case $$m=2.$$
  - Truncated Gamma ($\alpha , \beta$) model with distribution function
    $$\begin{aligned} F_X(x)=\frac{\gamma (\alpha , \beta x)}{\gamma (\alpha , \beta b)}, ~ 0<x\le b, ~\alpha ,\, \beta >0, \end{aligned}$$
    and Laplace transform
    $$\begin{aligned} M_X(-s) = \left( \frac{\beta }{\beta + s}\right) ^{\alpha } \frac{\gamma (\alpha , (\beta +s) b)}{\gamma (\alpha , \beta b)}, \end{aligned}$$
    where $\gamma (\alpha , x) = \int _0^x z^{\alpha -1} e^{-z} dz$ is the lower incomplete gamma function.
  - Truncated US-Pareto ($\alpha , \eta$) (Lomax) model with distribution function
    $$\begin{aligned} F_X(x)=\frac{1-\left( \frac{\eta }{\eta + x}\right) ^{\alpha }}{1-\left( \frac{\eta }{\eta + b}\right) ^{\alpha }}, ~ 0\le x\le b, ~\alpha ,\, \eta >0, \end{aligned}$$
    and Laplace transform
    $$\begin{aligned} M_X(-s) = \frac{\alpha (\eta s)^{\alpha } e^{\eta s}}{1-\left( \frac{\eta }{\eta + b}\right) ^{\alpha }}\left( \Gamma (-\alpha , \eta s) - \Gamma (-\alpha , (\eta +b) s)\right) , \end{aligned}$$
    where $\Gamma (\alpha , x) = \int _x^{\infty } z^{\alpha -1} e^{-z} dz$ is the upper incomplete gamma function.
- Case $m=3.$ Truncated generalized Gamma ($\alpha , \beta , \tau$) model with density and distribution function given by
  $$\begin{aligned} f_X(x)=\frac{\tau \, x^{\alpha \tau - 1} \beta ^{-\alpha \tau } e^{-\left( x/\beta \right) ^{\tau }}}{\gamma (\alpha , (b/\beta )^{\tau })}, ~ ~ F_X(x)=\frac{\gamma (\alpha , (x/\beta )^{\tau })}{\gamma (\alpha , (b/\beta )^{\tau })}, ~ 0<x\le b, ~\alpha ,\, \beta \, \tau >0, \end{aligned}$$
  and Laplace transform
  $$\begin{aligned} M_X(-s) = \sum _{k=0}^{\infty }\frac{(-\beta s)^k}{k!} \frac{\gamma (\alpha + k/\tau , (b/\beta )^{\tau })}{\gamma (\alpha , (b/\beta )^{\tau })}. \end{aligned}$$

In each case, the distribution parameters were determined using the method of moments for the given moment values set in the example above. For further details on claim size distributions and truncation see, for example, Albrecher et al. [Sec.3.3 & Ch.4] (2017).

**Fig. 3:**
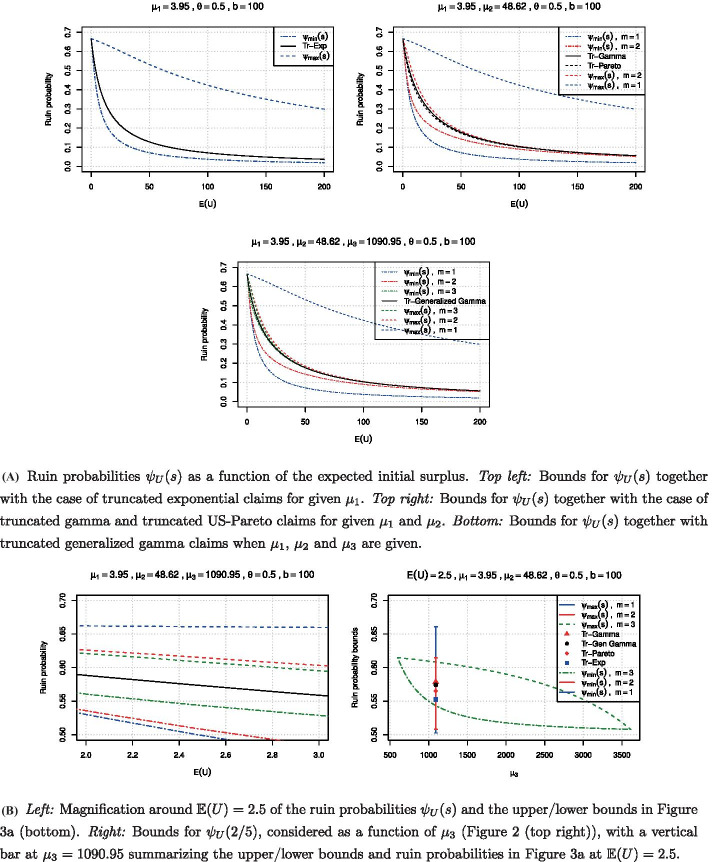
Numerical illustration for bounds of the ruin probability under exponentially distributed initial surplus

The results are given in Fig. 3a where the exact ruin probabilities obtained using (4) together with the general bounds are plotted as a function of the expected initial surplus $\mathbb {E}(U) = 1/s$ for the same set of parameters as above. In particular, *b* was selected in such a way that no strong truncation effect is present in the distributions. One sees that, for fixed $\mu _1$ only, the truncated exponential case is nicely between the sharp bounds. However, these bounds are very wide. When information about the second moment of *X* is included, the tightness of the bounds improves significantly. From (5), one would expect that to be the case only for small values of *s* where information about the first two moments provides a good approximation for the ruin probability. However, we can see that even for large values of *s* the improvement is considerable. The tightness of the interval for possible ruin probabilities becomes even more remarkable when the first three moments are fixed. This illustrates that in the context of ruin probabilities, the knowledge of the first few moments of the claim size distribution already provides a very accurate approximation. In a broader statistical context, for an account on reconstructions of arbitrary distributions from given moments, see e.g. Mnatsakanov (2008). Finally, for recent progress on the general probability level concerning criteria of moment-determinacy of distributions, see Yarovaya et al. (2020).

#### Remark 3.1

All results from this section can easily be generalized to the case where the initial surplus is assumed to be a mixture of exponential random variables. Indeed, consider a random initial surplus *O* with density

9 $$\begin{aligned} f_O(u) = \sum _{i = 1}^n q_i k_i e^{-k_i\cdot u}, \end{aligned}$$

with $0<q_i < 1, \sum _{i = 1}^n q_i = 1$ and $k_i > 0$ for $i = 1, \ldots , n.$ Then

10 $$\begin{aligned} \psi _{O} := \mathbb {E}(\psi (O))&= \sum _{i=1}^n q_i \int _0^{\infty } \psi (u) k_i e^{-k_i u} du = \sum _{i = 1}^n q_i \cdot \psi _U(k_i) = \sum _{i = 1}^n q_ik_i \cdot \widehat{\psi }(k_i) \nonumber \\&= 1 - \sum _{i=1}^n q_i k_i\, \frac{c-\lambda \mu }{ck_i - \lambda (1-M_X(-k_i))} \end{aligned}$$

Since Proposition 3.2 applies to any value $s>0,$ for every given set of *m* moment constraints and all $k_i >0, i = 1,\ldots , n,$ we obtain $\psi ^{\min }_U(k_i) \le \psi _U(k_i) \le \psi ^{\max }_U(k_i).$ Therefore,

$$\sum _{i=1}^n q_i \psi ^{\min }_U(k_i) \le \psi _O \le \sum _{i=i}^n q_i \psi _U^{\max }(k_i).$$

Consequently, when *U* is a mixture of exponential random variables, the lower and upper bounds for the expected ruin probability are linear combinations of the respective upper and lower bounds for the ruin probability with exponentially distributed initial surplus.

Note that for obtaining these sharp bounds, one still needed to reduce the expressions to purely exponential components so that the bounds on Laplace transforms can be used. For more general assumptions on *U* (like a general phase-type distribution) that link cannot be carried over in such a direct way. In the next section, we will, however, study the case of Erlang(*k*) distributed *U* in more detail, which is of particular interest, as for large *k* a deterministic initial surplus level can be approximated.

## Erlang Distributed Initial Surplus

A natural extension of exponentially distributed random surplus is now to consider Erlang distributed initial surplus. Concretely, consider *U* to be an Erlang(*k*, *s*) random variable $E_k$ with density

$$\begin{aligned} f_{E_k}(x) = \frac{1}{(k-1)!}s^k x^{k-1} e^{-s x}~\text { for } k\ge 1,\, s> 0,\, x>0, \end{aligned}$$

We then get

$$\begin{aligned} \psi _E(k,s):= \mathbb {E}(\psi (E_k))&=\int _{0}^{\infty }\psi (u)\frac{s^k}{(k-1)!}u^{k-1}e^{-s u}du =1+\frac{(-s)^{k}}{(k-1)!}\frac{\partial ^{(k-1)}}{\partial s^{(k-1)}}\widehat{\phi }(s). \end{aligned}$$

**Fig. 4:**
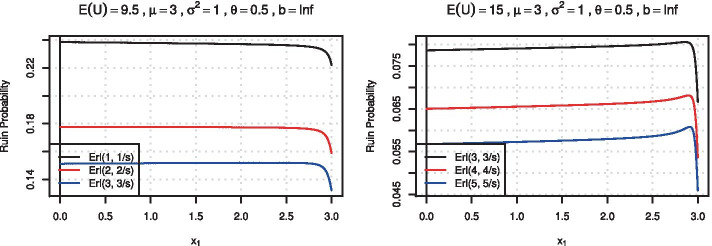
Ruin probabilities for Erlang(*k*, *k*/*u*) distributed surplus as a function of $x_1$ for $\mu =3$, $\sigma ^2=1$, $\theta =0.5$ and $\mathbb {E}(U)$ as specified

Here $\widehat{\phi }(s) = 1/s-\widehat{\psi }(s)$ denotes the Laplace transform of the survival probability of the classical Cramér-Lundberg risk process and we observe that its derivatives w.r.t. the Laplace argument lead to an explicit expression for the case of random Erlang-distributed initial surplus. We focus here on the classical Schmitter setting with fixed mean and variance of the claim size distribution. In Fig. 4 we depict the ruin probabilities for Erlang(*k*, *s*) distributed initial surplus for 2-point distributions as a function of $x_1$ for a given mean and variance, for two expected surplus levels.

In contrast to the exponential case $(k=1)$, there is unfortunately no direct relation between the optimization problem and the minimization (maximization) of the Laplace transform of the ruin probability. What we obtain is in fact an expression in terms of its $(k-1)$-th derivative (with $\partial ^{(0)}/\partial s^{(0)}\hat{\phi }(s) = \hat{\phi }(s)$). For example, for $k=2$ we get

$$\begin{aligned} \psi _E(2,s)&= 1+s^2\frac{\partial }{\partial s}\hat{\phi }_u( s) =1+s^2\frac{\partial }{\partial s}\frac{c-\lambda \mu }{c s-\lambda (1-M_X(-s))}\\ {}&=1-s^2\frac{c+\lambda \frac{\partial }{\partial s}M_X(-s)}{c-\lambda \mu }\bigg (\frac{c-\lambda \mu }{c s-\lambda (1-M_X(-s))}\bigg )^2,~\text { }s>0. \end{aligned}$$

Thus, for a fixed parameter *s*, in order to maximize our ruin probability, we need to minimize an expression that depends on both the Laplace transform of *X* and its first derivative.

Since the variance of a Erlang distribution goes to 0 as $k\rightarrow \infty$, one particular motivation to consider Erlang distributed initial surplus is as a tool to approximate the case of deterministic initial surplus, as in fact one has $\psi _{E}(k,s) \rightarrow \psi (u)$ as $k\rightarrow \infty$. The approximation $\psi (u) \approx \psi _E(k,s)$, or *Erlang smoothing*, was considered in Asmussen et al. (2002) as a numerical scheme to approximate the finite horizon ruin probability by replacing the deterministic time horizon *T* by an “standarized” Erlang(*k*, *k*/*T*) random variable, which for $k\rightarrow \infty$ becomes exact (see Asmussen and Albrecher [Ch.IX.8] (2009) for a more general discussion, as well as Stanford et al. (2005), Carr (1998) and Kyprianou and Pistorius (2003) for applications of this approach to other fields). Concerning the convergence rate with increasing *k*, for our context of random initial surplus one can adapt Theorem 6 of Asmussen et al. (2002) in a straight-forward way to obtain the following result:

### Proposition 4.1

Let $u>0$ be the expected initial surplus and let $E_k$ denote the Erlang distribution with shape parameter *k* and mean *u*. Then $\psi _{E}(k,s) \rightarrow \psi (u)$ as $k\rightarrow \infty .$ More precisely, for some constant *C*

11 $$\begin{aligned} \psi _E(k,k/u) = \psi (u) + \frac{C}{k} + O(k^{-2}). \end{aligned}$$

As already suggested in Asmussen et al. (2002), a further improvement of accuracy for fixed *k* can be obtained by Richardson extrapolation. This is a general method (see e.g. Press et al. (2007) for details) for computing an abstract quantity *y* (it could be an integral, a derivative, etc.) accurately using a sequence $y_k \rightarrow y$ for which the convergence rate is known,

$$y_k = y + \frac{c_1}{k} + \frac{c_2}{k^{1+\epsilon }}+\ldots ,$$

where $c_1$ is typically unknown but can be eliminated. In fact, setting $\tilde{y}_k = (k+1) y_{k+1} - ky_k,$ we get that $\tilde{y}_k \rightarrow y$ and one obtains an improved approximation of convergence rate $O(k^{-1-\epsilon }).$

Translated into our context, we then get

12 $$\begin{aligned} \psi (u) \approx (k+1)\,\psi _E(k+1, (k+1) / u) - k\,\psi _E(k, k / u), \end{aligned}$$

with an error rate of order $1/k^2.$

**Fig. 5:**
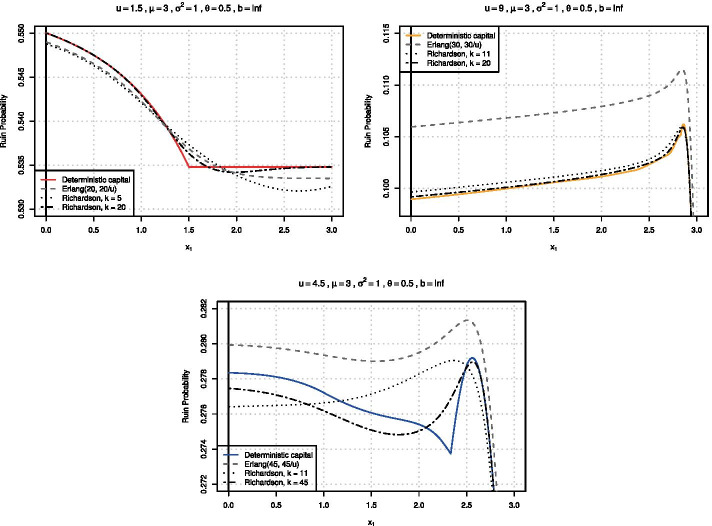
Ruin probabilities as a function of $x_1$ for $\mu =3$, $\sigma ^2=1$, $\theta =0.5$ and three levels of initial surplus *u* (expected surplus $\mathbb {E}(U)=1/s$, respectively)

For an illustration of the method, consider the same example as in Fig. 1, namely the set of 2-point distributions with mean $\mu =3,$ variance $\sigma ^2 = 1,$ and safety loading $\theta = 0.5.$ Figure 5 shows the results of the approximation. One observes that the approximation of the deterministic case via the randomized initial surplus is quite satisfactory already for $k=11$. The numerical approximation works well even for intermediate values of the initial surplus for which the ruin probability (and its kink) is difficult to approximate. In order to also reproduce the particular shape of that curve, higher values of *k* are however needed. It is worth to note the tremendous improvement when employing Richardson extrapolation for larger values of *u* (cf. the graph for $u=9$).

### Remark 4.1

Analogous to the exponential initial surplus case, one can obtain an expression for the ruin probability in terms of moments of *L*. Concretely,

$$\begin{aligned} \psi _E(k,s)&= \int _{0}^{\infty }\psi (u)\frac{s^k}{(k-1)!}u^{k-1}e^{-s u}du=\int _{0}^{\infty }\psi (u)\frac{s^k}{(k-1)!}\left( (-1)^{k-1}\frac{\partial ^{k-1}}{\partial s^{k-1}}e^{-s u}\right) du \\&= \frac{s (-s)^{k-1}}{(k-1)!}\frac{\partial ^{k-1}}{\partial s^{k-1}} \int _{0}^{\infty }\psi (u)e^{-s u} du \\&= \frac{s (-s)^{k-1}}{(k-1)!}\frac{\partial ^{k-1}}{\partial s^{k-1}} \sum _{j=0}^{\infty } \frac{(-s)^j}{j!} \int _{0}^{\infty }u^j\mathbb {P}(L>u) du\\&= \frac{s (-s)^{k-1}}{(k-1)!}\frac{\partial ^{k-1}}{\partial s^{k-1}} \sum _{j=0}^{\infty } \frac{(-s)^{j}}{(j+1)!} \,\mathbb {E}(L^{j+1}) \\&= \sum _{j\ge k-1} (-1)^{j+k-1}\frac{s^{j+1}}{(j+1)!}{j \atopwithdelims ()k-1} \mathbb {E}(L^{j+1}). \end{aligned}$$

Therefore, one might try to understand the behavior of the $\psi _E(k,s)$ by analyzing the first, say, two terms of the previous series to obtain the approximation

$$\psi _E(k,s) \approx \frac{s^k}{k!}\mathbb {E}(L^k) - \frac{s^{k+1}}{(k+1)!}k\mathbb {E}(L^{k+1}).$$

In the exponential case $k=1$, as the second moment of *l* involves the first three moments of *X*, this means that for given $\mu _1,\mu _2$ one could infer about the behavior of $\psi _U(s)$ by simply analyzing the first non-given moment, i.e. $\mu _{3}$. We see that the same line of reasoning applied to the Erlang(*k*) case needs the ($k+2$)-th moment of *X*, already for the above first two terms. This is unfortunate, as the deterministic $\psi (u)$ will only be obtained for $k\rightarrow \infty$, and we see that even in this simple approximation higher-order moments of *X* already play a crucial role. This is in particular the case for moderate values of $\mathbb {E}(U)$, and in those cases we have indeed seen in the graphs above that a good approximation of the deterministic case needed large values of *k*.

### Remark 4.2

When the goal is to approximate a deterministic initial surplus level, combinations of exponentials (i.e. densities of the form (9) but with $q_i\in {\mathbb R}$, $\sum _{i = 1}^n q_i = 1$) could a priori also be candidates for *U*, as that class is dense in the class of all distributions on the positive halfline, see e.g. Dufresne (2007)). Unfortunately, apart from the fact that an enormous number *n* will be needed for a reasonable approximation of a deterministic *u*, the differing signs of $q_i$ in (10) then also do not allow to identify extremal distributions as in Section 3.

## Conclusion

In this paper we showed how randomization can be used to provide a solution to the Schmitter problem in ruin theory and its extension to higher moments. Linking this problem with established results in the theory of *m*-convex stochastic orders, we provided sharp bounds for the ruin probability under the assumption of an exponential initial surplus. For the more general case of Erlang distributed initial surplus, such analytical sharp bounds are not within reach. However, we showed how the deterministic classical case can be approximated by the simple expressions of the randomized case using Erlangization.

## Footnotes

1 Note that in this paper we use the notation $\le _{m-cx}$ to denote *m*-convexity whenever the first *m* moments are available and not $(m-1)$ as it is standard in the *m*-convex risk literature.
